# Improving hospital quality risk-adjustment models using interactions identified by hierarchical group lasso regularisation

**DOI:** 10.1186/s12913-023-10423-9

**Published:** 2023-12-15

**Authors:** Monika Ray, Sharon Zhao, Sheng Wang, Alex Bohl, Patrick S. Romano

**Affiliations:** 1 Division of General Internal Medicine, School of Medicine, University of California, Davis, Sacramento, California USA; 2 Center for Healthcare Policy and Research, University of California, Davis, Sacramento, California USA; 3 Mathematica Inc., Princeton, New Jersey USA

**Keywords:** Hierarchical group lasso regularisation, Interaction effects, Risk-adjustment models, Hospital inpatient quality indicators

## Abstract

**Background:**

Risk-adjustment (RA) models are used to account for severity of illness in comparing patient outcomes across hospitals. Researchers specify covariates as main effects, but they often ignore interactions or use stratification to account for effect modification, despite limitations due to rare events and sparse data. Three Agency for Healthcare Research and Quality (AHRQ) hospital-level Quality Indicators currently use stratified models, but their variable performance and limited interpretability motivated the design of better models.

**Methods:**

We analysed patient discharge de-identified data from 14 State Inpatient Databases, AHRQ Healthcare Cost and Utilization Project, California Department of Health Care Access and Information, and New York State Department of Health. We used hierarchical group lasso regularisation (HGLR) to identify first-order interactions in several AHRQ inpatient quality indicators (IQI) - IQI 09 (Pancreatic Resection Mortality Rate), IQI 11 (Abdominal Aortic Aneurysm Repair Mortality Rate), and Patient Safety Indicator 14 (Postoperative Wound Dehiscence Rate). These models were compared with stratum-specific and composite main effects models with covariates selected by least absolute shrinkage and selection operator (LASSO).

**Results:**

HGLR identified clinically meaningful interactions for all models. Synergistic IQI 11 interactions, such as between hypertension and respiratory failure, suggest patients who merit special attention in perioperative care. Antagonistic IQI 11 interactions, such as between shock and chronic comorbidities, illustrate that naïve main effects models overestimate risk in key subpopulations. Interactions for PSI 14 suggest key subpopulations for whom the risk of wound dehiscence is similar between open and laparoscopic approaches, whereas laparoscopic approach is safer for other groups. Model performance was similar or superior for composite models with HGLR-selected features, compared to those with LASSO-selected features.

**Conclusions:**

In this application to high-profile, high-stakes risk-adjustment models, HGLR selected interactions that maintained or improved model performance in populations with heterogeneous risk, while identifying clinically important interactions. The HGLR package is scalable to handle a large number of covariates and their interactions and is customisable to use multiple CPU cores to reduce analysis time. The HGLR method will allow scholars to avoid creating stratified models on sparse data, improve model calibration, and reduce bias. Future work involves testing using other combinations of risk factors, such as vital signs and laboratory values. Our study focuses on a real-world problem of considerable importance to hospitals and policy-makers who must use RA models for statutorily mandated public reporting and payment programmes.

## Introduction

In health care, risk-adjusted (RA) outcome measures are widely used to compare performance across health care organisations. Stakeholders such as the Centers for Medicare & Medicaid Services (CMS), use these measures to rate hospitals (e.g., Care Compare [14]), to inform accreditation processes, to assign financial rewards and penalties (e.g., Hospital-Acquired Conditions Reduction Program), and to drive quality improvement activities. As the lead federal agency tasked with improving the safety and quality of American health care, the Agency for Healthcare Research and Quality (AHRQ) develops and maintains a suite of measures called the AHRQ Quality Indicators (QIs) [3, 5]. AHRQ’s Inpatient Quality Indicators (IQIs) focus on risk-adjusted mortality among patients hospitalised with life-threatening conditions (e.g., heart attack, pneumonia, stroke) and invasive surgical procedures (e.g., abdominal aortic aneurysm repair, hip fracture repair), while the Patient Safety Indicators (PSIs) focus on potentially preventable complications of hospital care.

Several AHRQ QIs employ stratified RA models because their denominator populations are heterogenous while their numerator definitions are identical. Although stratified models represent a valid approach for estimating heterogeneous effects [11], the resulting models have highly variable performance due to extremely low event rates and limitations of stratum-specific feature selection. A better and interpretable solution to the problem of population heterogeneity in RA models would involve including linear interactions in the model [25, 35]. Furthermore, predictive models for health outcomes often have poor calibration, potentially due to interactions that are ignored by standard methods [21]. Surgical outcome reports have shown that these interactions can be either synergistic or antagonistic [10, 23, 24, 31], yet they are often overlooked in RA models. Some vendors have developed risk-adjustment approaches that pre-specify interactions, such as 3M’s All Patient Refined Diagnosis Related Groups (APR-DRGs) [1], but public agencies such as AHRQ [5] have stopped using proprietary tools due to their cost and opaqueness.

Least absolute shrinkage and selection operator (LASSO) is a feature selection method for developing risk models that relies upon penalised regression, and shrinking coefficients to zero [41], which offers important benefits over stepwise selection approaches based on *p* values [38]. However, it does not have a mechanism to automatically identify all pairwise interactions but rather depends on manual specification of each interaction to test. This procedure leads to spurious interactions being forced into the model or important interactions being omitted when there are several hundred covariates/dimensions as is the case with healthcare datasets. Most standard implementations of regularised models fail to satisfactorily address two issues that characterise healthcare data - (a) the quadratic explosion of interactions, and (b) the presence of categorical variables with multiple levels of values, such as Medicare Severity Diagnosis Related Groups (MS-DRGs) and Major Diagnostic Categories (MDCs). Hierarchical Group-LASSO Regularisation (HGLR) is a novel feature selection method for identifying *first order interactions* that enforces *strong hierarchy* based on regression trees [26]. HGLR sets up main effects and interactions via groups of variables, and then performs feature selection via group-LASSO, which is a generalisation of the LASSO for selecting groups of variables [42]. Both HGLR and LASSO are regularisation methods that shrink non-informative features’ coefficients to zero, thereby removing them from the model. HGLR is attractive because (1) if there are no true interactions, then glinternet only selects the main effects, and (2) it is a linear model with complexity comparable to penalised regression. Therefore, it can handle problems with several thousand features and retain the interpretability of linear models.

Our aim was to investigate whether AHRQ’s stratified QI RA models could be replaced by composite RA models, using HGLR to select and estimate clinically meaningful and interpretable interactions. These AHRQ QIs represent a useful test case for this novel approach given their widespread use by federal and state health agencies, and other stakeholders such as employer coalitions [40], for ranking hospitals and assigning rewards and financial penalties. IQI 11, Abdominal Aortic Aneurysm (AAA) Repair Mortality Rate, is stratified into four groups based on the type of AAA repair (open vs. endovascular) and AAA rupture status. IQI 09, Pancreatic Resection Mortality Rate, is stratified into two groups based on the absence or presence of a pancreatic cancer diagnosis. PSI 14, Postoperative Wound Dehiscence Rate, is stratified into two groups based on whether the salient abdominopelvic operation was performed by open or laparoscopic (non-open) approach. PSI 04, Death Rate among Surgical Inpatients with Serious Treatable Complications, is stratified into five groups based on the type of triggering complication, but this PSI was not analysed as it is being currently redesigned. The QI stratified models have demonstrated variable performance; for example, the currently reported C-Statistics for IQI 11 stratified models range from 74% to 87% [4, 7]. Some stratified models are also limited by rare events; for example, the current version of PSI 14 has just 101 numerator events among 567,439 denominator encounters in the non-open stratum. Our approach involved re-estimating these stratified models using HGLR to identify important interactions, and comparing the results with traditional LASSO selection of main effects alone.

## Methods

### Data

We analysed hospital stays of adults (Age $\ge$ 18 years) using de-identified inpatient discharge data from the 14 State Inpatient Databases (SID), Healthcare Cost and Utilization Project (HCUP), Agency for Healthcare Research and Quality [2], the California Department of Health Care Access and Information (HCAI), and the New York State Department of Health (NYSDOH). Our IQI data included 2016, 2017, and 2018 records from Arizona, California, New York, Maryland, Iowa, Massachusetts, New Jersey, New Mexico, Florida, Kentucky, Maine, Minnesota, Nebraska, Nevada, Vermont and Washington. Our PSI data included 2019, 2020, and 2021 records from California, New York, Maryland, and Florida. In these data, each row refers to an inpatient encounter and columns include encrypted hospital and patient identifiers, MS-DRGs, MDCs, admission source and type, ICD-10-CM diagnosis codes and ICD-10-PCS procedure codes etc. [2, 6].

### Analytic approach

#### Population

First, we ran AHRQ’s v2021 IQI and v2022 PSI software on the inpatient discharge data to identify the population (denominator) and adverse events (numerator) for each indicator [6], based on the ICD-10-CM diagnosis and ICD-10-PCS procedure codes in the data, and the corresponding ‘present on admission’ (POA) diagnosis flags and procedure dates. AHRQ’s Clinical Classification Software Refined (CCSR) was applied to all POA and POA-exempt diagnoses [8], following CMS’ approach [13], to adjust only for clinical conditions that were present on admission to the hospital. The CCSR software groups all diagnosis codes into 540 binary categorical variables.

We then combined multiple population strata in IQI 09, IQI 11, and PSI 14 into a single composite model for each indicator, with the same numerator outcome, and represented the strata in a new variable named IQIStrata or PSIStrata. IQIStrata had four levels (Open Ruptured, Endo Ruptured, Open Unruptured, Endo Unruptured) in IQI 11 and two levels (With Cancer, Without Cancer) in IQI 09. PSIStrata had two levels (Open And Non-Open) in PSI 14. The input features for the IQI models were DXCCSR binary categorical variables, 4 age categories, gender, hospital transfer status, and the IQIstrata. The features for PSI 14 included MS-DRGs collapsed into Modified DRGs by combining adjacent MS-DRGs with or without comorbidities or complications, 25 MDCs, age categories, sex, do-not-resuscitate (DNR) status, hospital transfer status, HCUP Elixhauser Comorbidity Software Refined [20], and PSIStrata. The MS-DRGs and MDCs were generated using CMS’s v39.1 MS-DRG Classifications software [12]. The outcome for the IQIs is mortality and that for PSI 14 is wound dehiscence.

#### Data preprocessing

In order for these models to meet AHRQ/CMS needs, we had to address several practical challenges, related to the clinical context and computing resources. Due to the large data set and sparsity of PSI 14 events, we undersampled the data to have 10,000 records for feature selection and removed some Modified DRG variables that represent complications of inpatient care (e.g., tracheostomy, abdominal wall hernia procedure) or provide no useful clinical information (e.g., "Operating Room (OR) procedure unrelated to principal diagnosis"). Random undersampling of non-event cases was performed using the Random Over-Sampling Examples (ROSE package in R) method [29, 30]. These sampling strategies do not pertain to the IQIs as their event rates were much higher. We also removed extremely low-frequency covariates before feature selection, as selection of these features would have led to convergence problems during subsequent maximum likelihood estimation in LASSO.

#### Feature selection phase

We used the following R packages - *glinternet* for HGLR, *glmnet* for LASSO, *logistf* for Firth’s logistic regression [18, 37], *precrec* [39] for precision-recall performance metrics. The data were split into 80-20% training-test sets. Feature selection was performed using ten-fold cross-validation (CV). The final model along with the corresponding regularisation parameter (lambda) value was chosen based on model’s performance (cross-validation error rate/area under the receiver operating characteristic (AUC)) on the training set, following standard machine learning protocols.

#### Final risk-adjusted models

As both AHRQ and CMS release their RA models as logistic regression models for transparency and interpretability, we reported the performance of logistic regression models using either the HGLR-selected (with interactions) or LASSO-selected (without interactions) features on the 20% test set. We report the area under the receiver operating characteristic (AUROC or C-Statistic) and the area under the precision-recall curve (AUPRC) as measures of discrimination in large imbalanced datasets. We used Firth’s logistic regression for PSI 14 due to the large standard errors for the parameters of interest, given the extremely low event rate and sparse cells. Firth’s method is specifically designed for these scenarios and is a suitable alternative to standard logistic regression for rare events [18, 37]. Model calibration was evaluated using calibration belts, where the x-axis represents the predicted risk of the event and the y-axis represents the observed risk. Finazzi et al. developed a method to assess calibration of models for binary outcomes called the calibration belt [32, 33]. A calibration belt graphically shows the confidence band around the calibration curve and is constructed from a function based on a generalisation of Cox’s seminal work [15]. This method is a useful alternative to HL calibration plots [33] as it helps in understanding the model’s behaviour without binning samples and is particularly important in large datasets [17, 34]. High *p*-values indicate that the difference between the observed and predicted scores is negligible.

## Results

Table 1 depicts the population characteristics for the all the AHRQ QIs, and the positive event rates across them. The IQI 11 sample was predominantly male (71.4-80.4%) with an average age of 73.7 years. By contrast, IQI 09 had roughly equal numbers of men and women in each stratum and a mean age of 62.8 years. The IQI 11 strata for open and endovascular treatment of ruptured aneurysms were dominated (66.9-69.3%) by emergency admissions with long LOS (mean 8.3-12.4 days) and high inpatient mortality (20.9-38.7%), while the other strata were dominated by elective admissions with low inpatient mortality (0.8-5.7%). The PSI 14 cohort was predominantly female (54.4-58.8%) with younger mean age (55.0-59.7 years). Most importantly, PSI 14 had an extremely low event rate.

**Table 1:** Characteristics of IQI 11 abdominal aortic aneurysm repair mortality rate and IQI 09 pancreatic resection mortality rate

AHRQ QI	Event	Popul.(N)	Mean Age	Male	Mean LOS	Admit Type
	rate(%)		(yrs)	(%)	±Std.(Days)	(%)
**IQI 11**						
OPEN RUPT	38.73	1,322	72.4±9.8	75.0	12.4±14.5	Em: 69.3
						U:16.6
						El:5.0
						O:9.1
ENDO RUPT	20.94	2,082	74.2±10.0	78.3	8.3±10.8	Em:66.9
						U:18.3
						El:8.2
						O:6.7
OPEN UNRUP	5.73	4,154	69.9±9.0	71.4	9.9±9.0	Em:14.8
						U:7.8
						El:75.1
						O:2.3
ENDO UNRUP	0.84	29,611	74.3±8.7	80.4	2.9±4.4	Em: 10.9
						U:6.8
						El:81.0
						O:1.3
**Composite**	3.86	37,169	73.7±9.0	79.1	4.27±6.8	Em: 16.6
						U: 7.9
						El: 73.6
						O: 2.0
**IQI 09**						
WITH	2.11	12,177	66.5±11	52.0	11.2±9.6	Em:7.6
CANCER						U: 5.2
						El:85.7
						O: 1.6
WITHOUT	2.49	10,017	58.2±15.2	47.8	10.9±13.1	Em: 9.7
CANCER						U: 5.9
						El:80.4
						O: 4.1
**Composite**	2.28	22,194	62.8±13.8	50.1	11.1±11.3	Em: 8.5
						U: 5.5
						El: 83.3
						O: 2.7
**PSI 14**						
OPEN	0.24	530,751	59.7±16	45.6	8.7±12.9	NA
NON-OPEN	0.01	438,531	55.0±18	41.2	5.2± 8.5	NA
**Composite**	0.13	969,282	57.6±17	43.6	7.1±11.3	NA

*Abbreviations: RUPT *Ruptured, *UNRUP *Unruptured, *Em *Emergency, *U *Urgent, *El *Elective, *O *Other admission types, *Composite *non-stratified model

Table 2 shows the performance of the feature selection models, HGLR and LASSO, on the hold-out test set. The HGLR model has two-way interaction terms (interaction model) whereas the LASSO model (non-interaction model) does not. Table 3 shows the performance of the risk-adjusted models of the three composite outcomes estimated with either LASSO-selected or HGLR-selected features. For IQI 11, interactions accounted for about half of the entire feature set and including these terms did not adversely affect the model’s performance or cause convergence issues. While both HGLR and LASSO selected IQIStrata (the population stratum) as a main effect in IQI 11 model, HGLR was able to automatically identify several clinically important interaction effects involving IQIStrata. Similarly, HGLR was able to identify five clinically important interaction effects involving PSIstrata in the PSI 14 model, with little impact on model performance despite substantially fewer main effects in the model with HGLR-selected features.

**Table 2:** Feature selection model performance

AHRQ QI	Model	C-Stat	AUPRC
PSI 14 composite	HGLR	0.856	0.008
	LASSO	0.853	0.008
IQI 11 composite	HGLR	0.917	0.407
	LASSO	0.910	0.427
IQI 09 composite	HGLR	0.761	0.126
	LASSO	0.762	0.129

**Table 3:** Risk-adjusted model performance

AHRQ QI	Features Set	C-Stat	AUPRC	Total No.
				Features
PSI 14 composite	HGLR ftrs	0.840	0.007	21
	LASSO ftrs	0.856	0.008	52
IQI 11 composite	HGLR ftrs	0.915	0.405	38
	LASSO ftrs	0.903	0.408	21
IQI 09 composite	HGLR ftrs	0.760	0.120	75
	LASSO ftrs	0.765	0.130	20

The HGLR feature set includes interactions while the LASSO feature set does not. *Abbreviation*: ftrs - Features

**Fig. 1:**
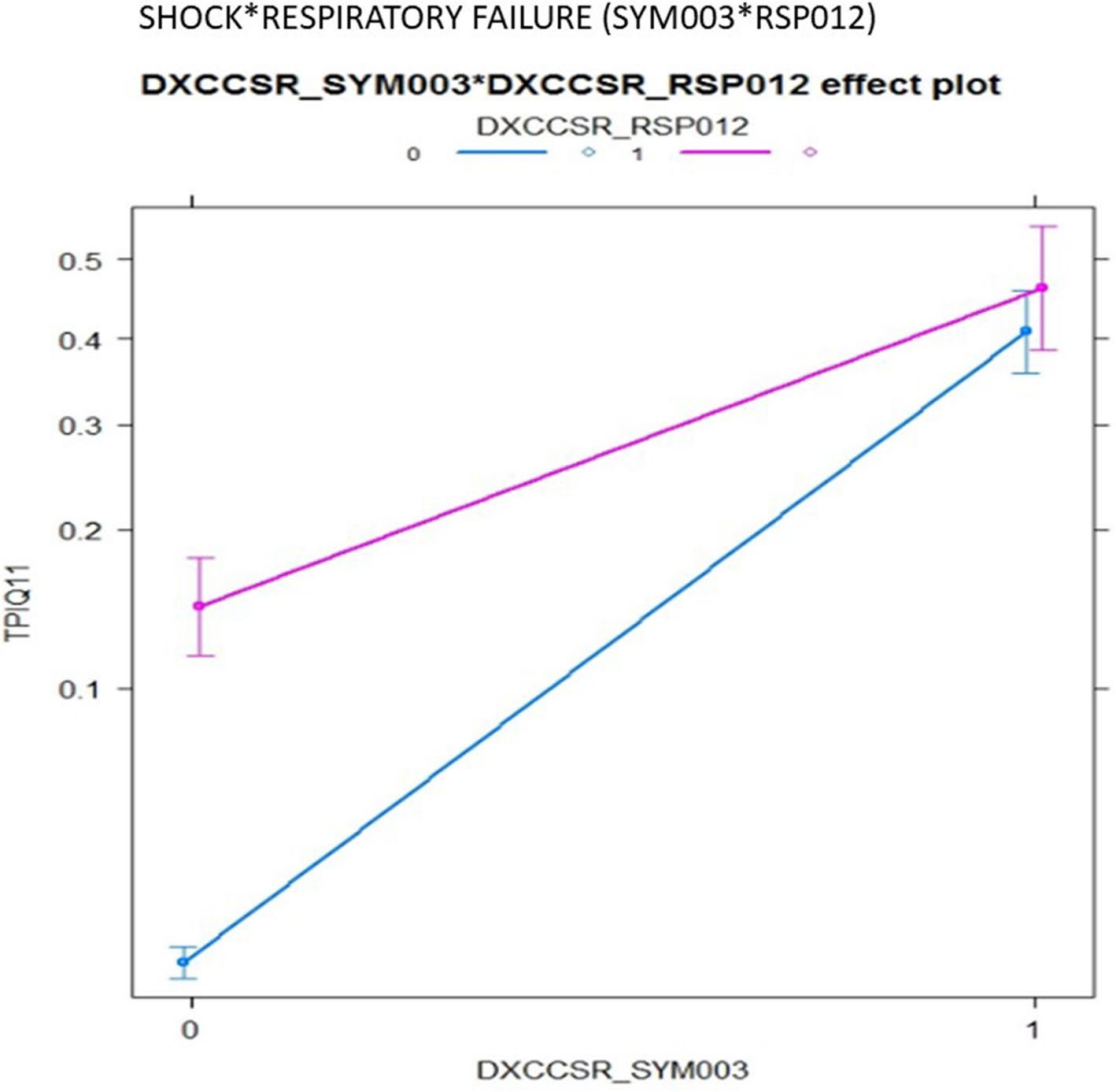
Interaction plot of shock and respiratory failure identified by HGLR for IQI 11 composite

**Fig. 2:**
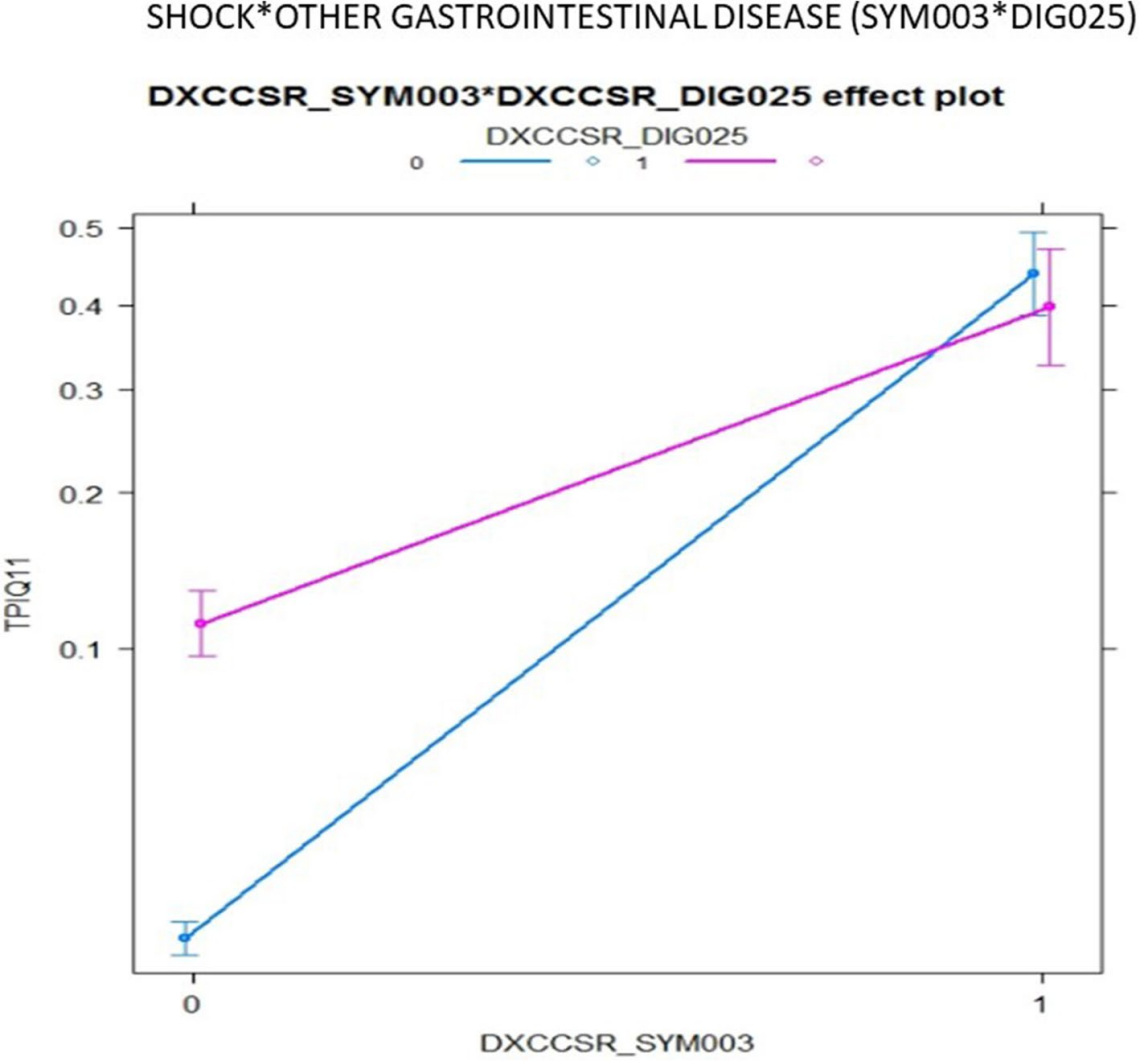
Interaction plot of shock and other gastrointestinal disease identified by HGLR for IQI 11 composite

**Fig. 3:**
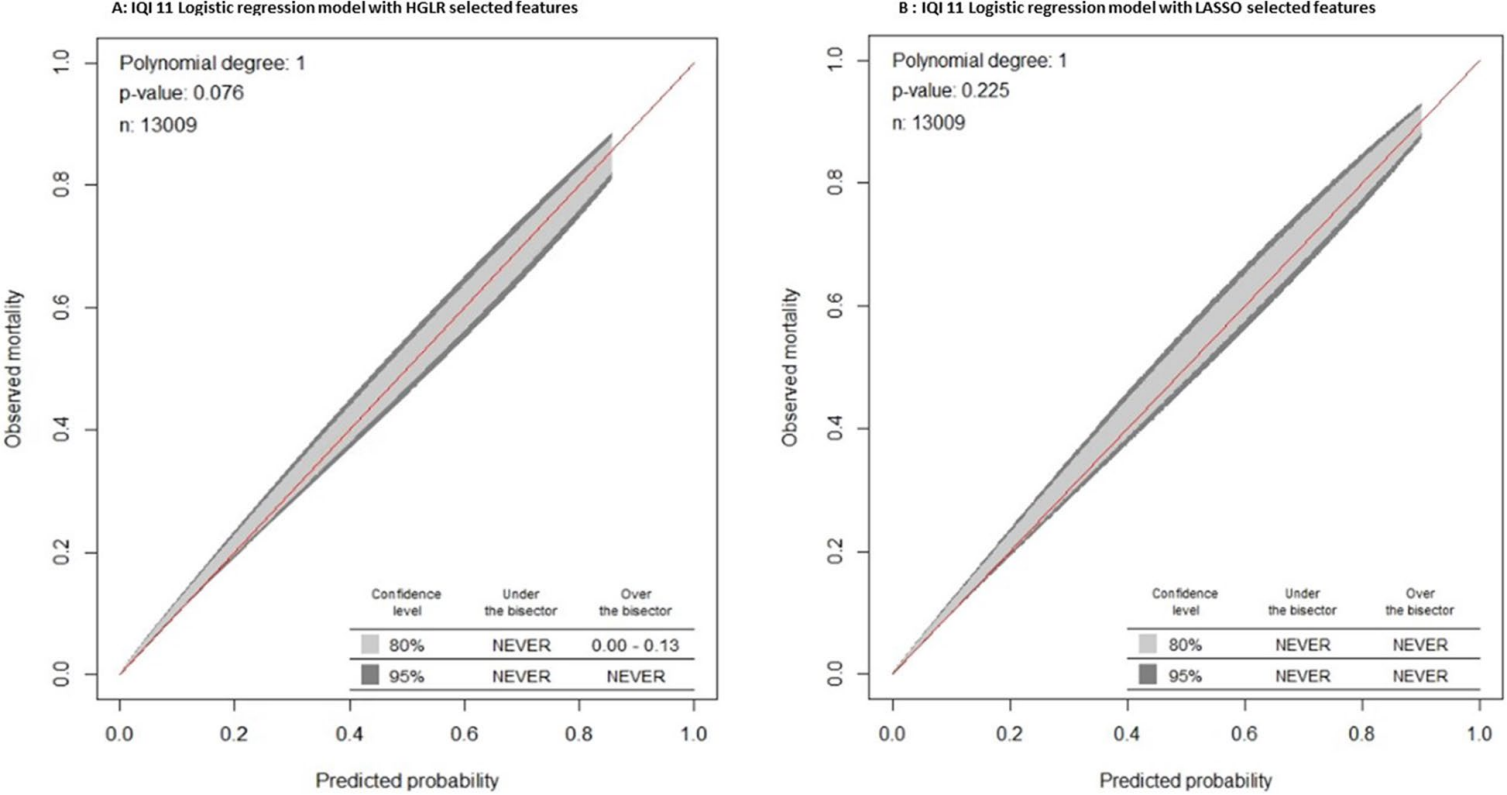
IQI 11 composite- Calibration belts of logistic regression models using **A**) HGLR or **B**) LASSO selected features

Table 4 shows the performance of stratified RA models with LASSO-selected features for comparison with the composite models shown in Table 3. Model discrimination using LASSO-selected features varies substantially across strata, from 0.689 to 0.834 for IQI 09, and from 0.683 to 0.801 for IQI 11. The Fig. 1 shows an interaction plot between shock and respiratory failure for IQI 11, demonstrating a significant negative interaction or antagonistic effect, with logistic parameter estimates of 0.871, 0.516, and -0.577 for shock, respiratory failure, and their two-way interaction, respectively. These estimates indicate that the presence of shock essentially negates the marginal effect of respiratory failure among patients undergoing AAA repair, such that the co-occurrence of both conditions is similar to having either condition alone. A negative interaction was also found between shock and gastrointestinal disease (Fig. 2). Interactions with IQIStrata generally showed larger relative effects in strata with lower baseline risk. The IQI 11 composite interaction model demonstrated slightly better discrimination (AUC, 0.915 versus 0.903) than the model with LASSO selected features, with similar calibration (Table 4; Fig. 3).

**Table 4:** Risk-adjusted model with LASSO features: stratified models performance

AHRQ QI	C-Stat	AUPRC
PSI 14 OPEN	0.774	0.008
PSI 14 NON-OPEN	0.826	0.000
IQI 11 OPEN RUPTURED	0.701	0.597
IQI 11 ENDO RUPTURED	0.763	0.444
IQI 11 OPEN UNRUPTURED	0.683	0.138
IQI 11 ENDO UNRUPTURED	0.801	0.061
IQI 09 with CANCER	0.689	0.081
IQI 09 without CANCER	0.834	0.212

**Fig. 4:**
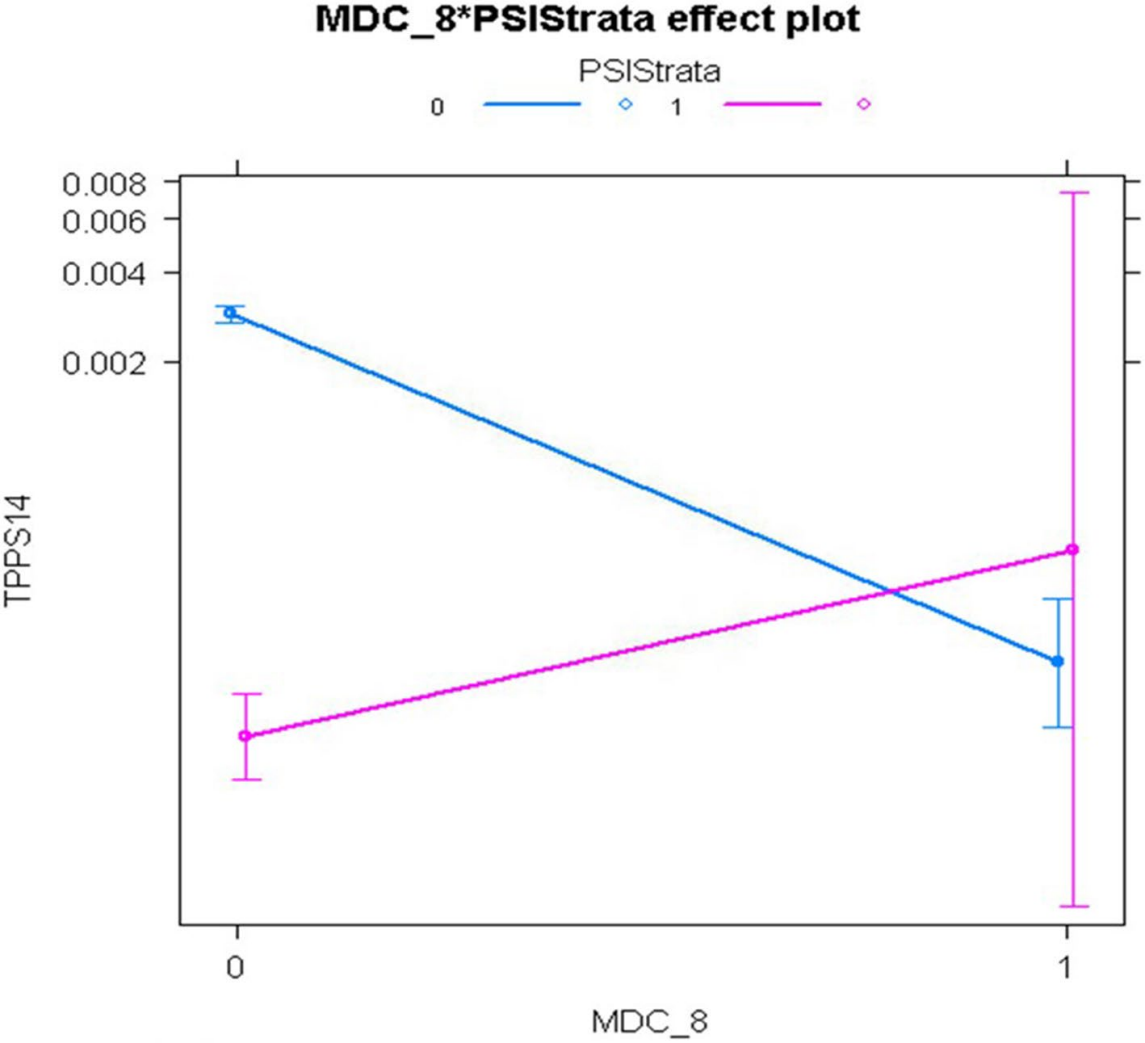
Interaction plot of MDC 08 (Diseases and Disorders of the Musculoskeletal System and Connective Tissue) and Population Strata identified by HGLR for PSI 14 composite

**Fig. 5:**
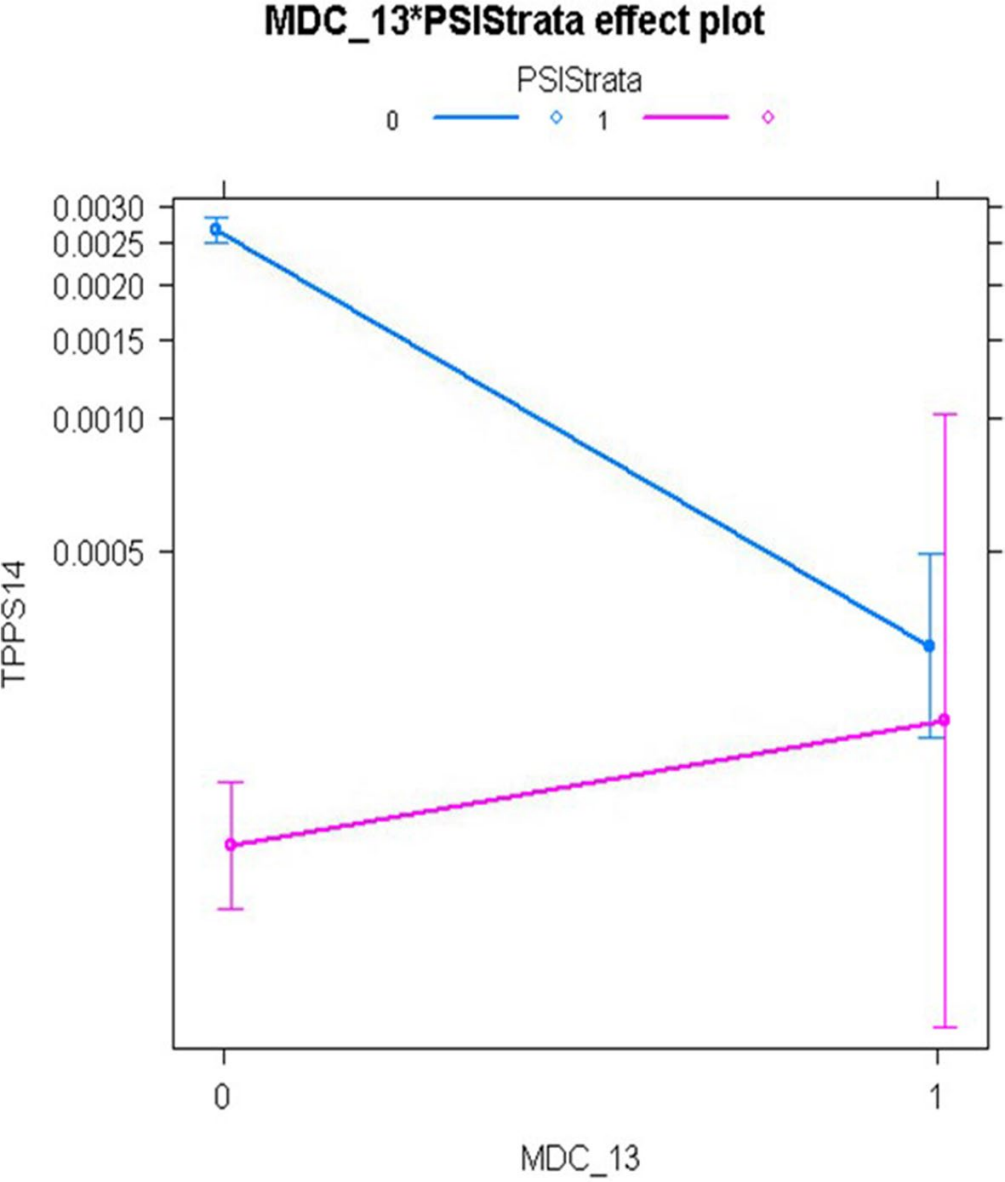
Interaction plot of MDC 13 (Diseases and Disorders of the Female Reproductive System) and Population Strata identified by HGLR for PSI 14 composite

**Fig. 6:**
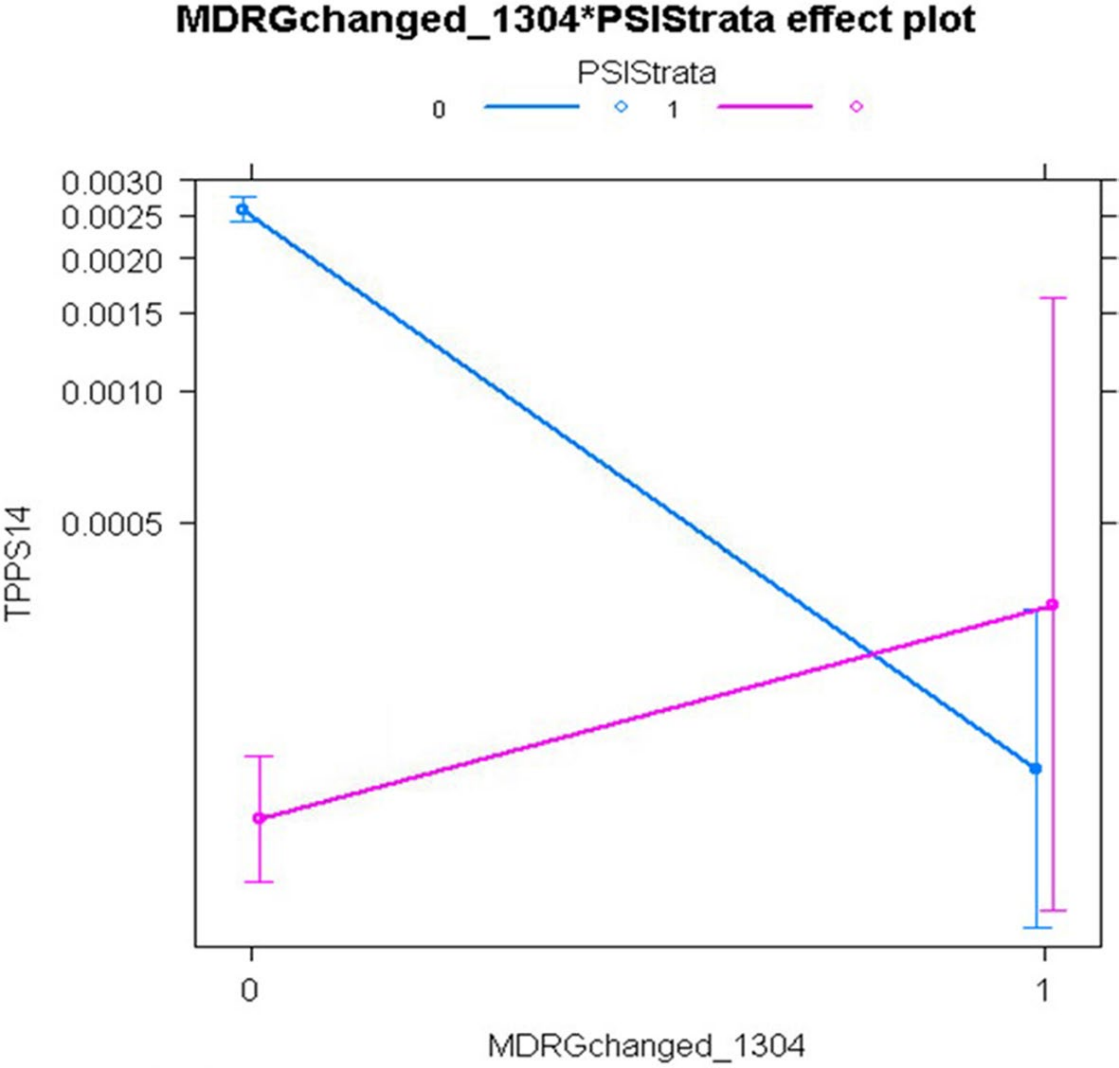
Interaction plot of Modified DRG 1304 (Uterine and Adnexa Procedure for Non-Malignancy) and Population Strata identified by HGLR for PSI 14 composite

**Fig. 7:**
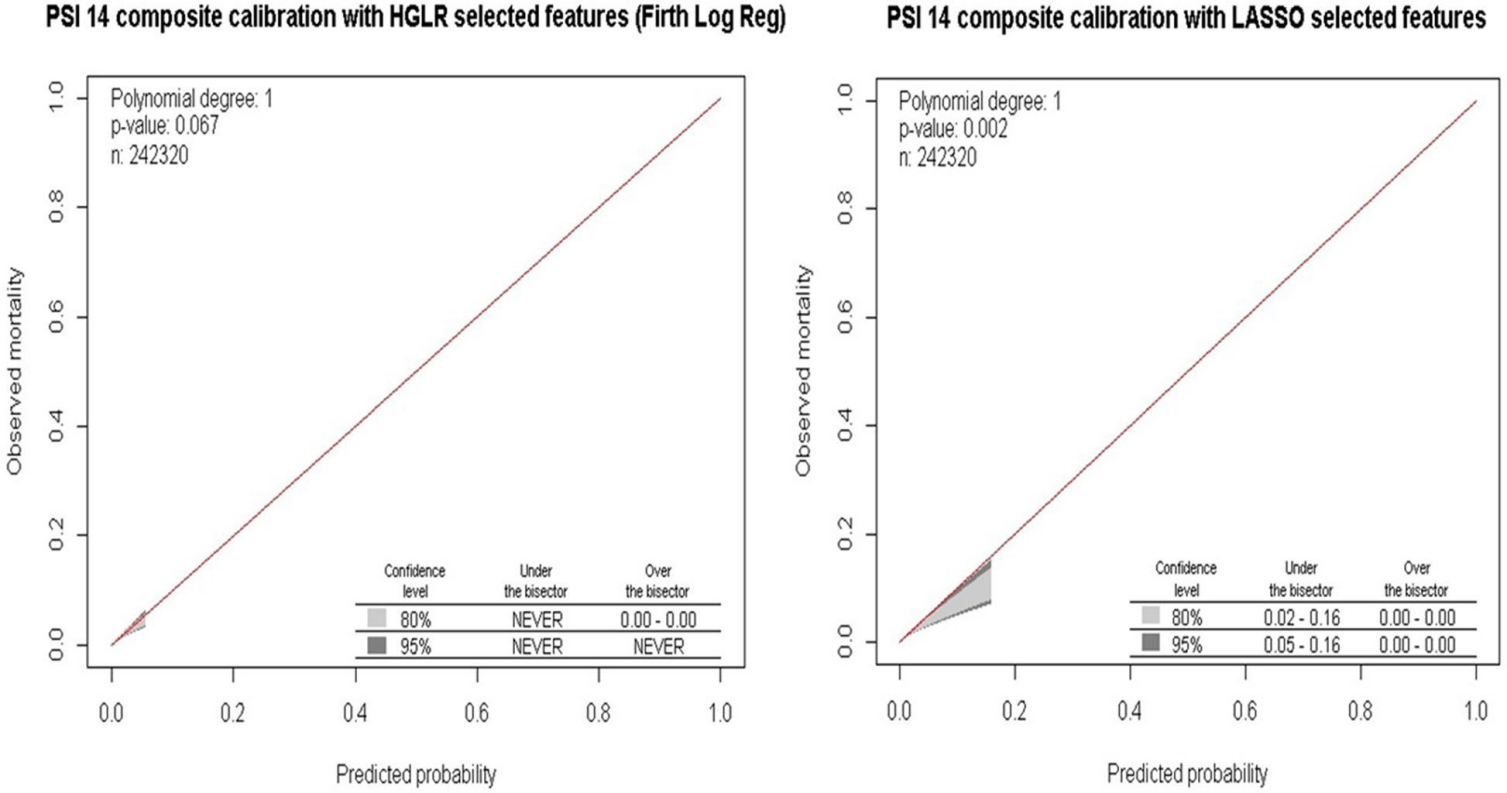
PSI 14 composite- Calibration belts of logistic regression models using **A**) HGLR or **B**) LASSO selected features

For PSI 14, the main effect of the non-open approach, relative to the open approach, was highly significant with adjusted odds ratios of 0.039 (95% confidence interval [CI], 0.028-0.055) in the non-interaction model and 0.047 (95% CI, 0.027-0.078) in the interaction model. However, the interaction model revealed that this beneficial effect of laparoscopic surgery was eliminated for patients in MDC 08 (Diseases and Disorders of the Musculoskeletal System and Connective Tissue), MDC 13 (Diseases and Disorders of the Female Reproductive System), and Modified DRG 1304 (Uterine and Adnexa Procedure for Non-Malignancy). Although LASSO selected over twice as many main effects as HGLR and achieved similar model discrimination as HGLR, it could not identify the interactions shown in Figs. 4, 5, and 6, which improved model calibration for higher risk patients as shown in Fig. 7 (goodness-of-fit *p*=0.067 for HGLR-selected features versus *p*=0.002 for LASSO-selected features).

## Discussion

This research was motivated by a practical problem in the development of RA models for high-stakes, policy-relevant applications, such as hospital quality measurement and payment. Specifically, model developers often either ignore clinically important two-way interactions, leading to prediction error for subpopulations that may cluster at specific sites, or estimate fully stratified models, thereby wasting degrees of freedom and limiting the ability to select a robust feature set for every stratum. We investigated an innovative solution to this challenge, using HGLR to estimate composite models instead of stratified models while identifying clinically important interactions representing a robust set of heterogeneous effects. Given several hundred features in these applications, it is infeasible to manually specify all possible two-way combinations, nor is it advisable to specify such a model due to issues that arise during feature selection or parameter estimation such as collinearity and quasi-completeness.

### Clinical significance

Analysis of IQI 11 revealed important findings with respect to the specific features selected. We were able to combine four strata and estimate a single, more robust model with interactions, which is more efficient and generates more easily interpreted estimates of effect heterogeneity. The most common pattern of the selected interactions was that comorbidities such as chronic kidney disease, pleural effusion, other lower respiratory tract disease, fluid and electrolyte disorders, heart failure and malnutrition were associated with markedly increased mortality among patients with unruptured aneurysms who were treated with endovascular repair, but had little marginal effect among patients with ruptured aneurysms for whom baseline mortality was very high. This pattern is clinically logical and consistent with prior literature on obesity [27], as comorbid conditions may have less relative impact on inpatient mortality in the setting of a catastrophic acute condition than with a stable chronic condition, such as an intact aneurysm. The exception to this pattern was for shock, which is a marker of the severity of acute aneurysm rupture.

Using HGLR, we were also able to identify several previously unsuspected but clinically meaningful interactions among comorbid conditions. When these interaction effects were positive or synergistic, as for hypertension and respiratory failure, they suggest combinations of risk factors that may merit special attention as part of perioperative care. More commonly, these interaction effects were negative or antagonistic, suggesting that the combination of interacting factors was associated with little or no marginal increase in risk, compared with having one of those factors alone. For example, the presence of shock at admission is an important predictor of postoperative death. Shock was found to interact with several other clinical factors (e.g., peripheral vascular disease, respiratory failure, other gastrointestinal disorders) such that the presence of shock virtually eliminated or reduced the marginal effect of the interacting factor on inpatient mortality. Failure to account for such important interactions may lead to overestimation of risk among patients with combinations of risk factors, and thus miscalibration of risk-adjustment models in portions of the risk distribution. Such miscalibration may bias risk-standardized outcome rates for specific facilities that attract patients with combinations of risk factors. Two-way interactions were also identified for IQI 09, but given the similar risk of inpatient death between patients with and without cancer undergoing pancreatic resection, these interactions were less clinically interesting than those in IQI 11 and PSI 14 models.

For PSI 14, the two strata have markedly different event rates, with a very low event rate in the non-open stratum. It is widely accepted that the risk of postoperative wound dehiscence is higher with the open approach than with the laparoscopic approach, but the HGLR-derived model suggests that this difference is unexpectedly heterogeneous, and that the laparoscopic approach may not confer benefit (in terms of postoperative wound dehiscence) for patients undergoing musculoskeletal operations, such as lumbar diskectomy, or operations on the female reproductive system, such as hysterectomy. This intriguing finding requires validation in other large data sets.

### Model performance

Since RA models are often used to evaluate healthcare entities using data beyond the reference population on which the model was estimated, it is important to evaluate the predictive and generalisation capabilities of the model. The ROC curve and the area under it show the degree of separability between the two classes (i.e., event-positive and event-negative patients). The AUPRC shows the predictive performance of the model and is helpful to understand models developed on imbalanced datasets, where the number of negative events far exceeds the number of positive events [16, 36, 39]. Our results show that combined models perform as well or better than stratified models, and that an automated approach to selecting linear interactions from an extremely large number of possibilities yields combined models with similar or better performance than traditional LASSO-based feature selection, limited to main effects. Notably, we found that HGLR never failed to converge, even when LASSO demonstrated convergence problems.

## Conclusion

Key strengths of this study include the large, diverse, and population-based sample of patients from numerous US states, and our focus on a real-world problem of considerable importance to hospitals, health care providers, researchers, and policy-makers who must use risk-adjustment models for statutorily mandated public reporting and payment programmes. HGLR has been used in various domains such as to analyse energy consumption in buildings in New York City [22], environmental effect of exposome on health [9], drug interactions from electronic health records and biomolecular data [28], and valuation of variable annuity portfolios [19], but it has not been used in health services research. Notably, the HGLR package has been implemented to be scalable to handle a large number of covariates and test all their interactions, and to be customisable to use multiple computing cores to reduce analysis time, which is an important consideration in the current era of big data and testing a large number of pairwise interactions. Limitations of this study will be addressed in future work by applying HGLR to other quality indicators, including unstratified models, as our work suggests that it may be advantageous to identify unsuspected interactions among clinical features. We will also test HGLR and other interaction methods using other risk factors (vital signs, laboratory values, etc.) and data from more U.S. states for assessing generalisability.

In risk-adjustment models for clinical events, it is often critical to include interactions accounting for synergistic and antagonistic effects. However, identifying such two-way interactions has been technically challenging, leading scholars to favour stratified models or to select a limited set of manually constructed interactions for evaluation. The former approach is inefficient and leads to models that are not directly comparable, as they are built on different populations and therefore do not support formal testing for heterogeneous covariate effects [11]. The latter approach is intuitively attractive for minimising the risk of spurious interactions, but it precludes discovering unsuspected interactions, and HGLR manages the risk of false discovery through multiple cross-validation and testing in a hold-out sample. In this application to widely used, high-stakes RA models, we have shown that HGLR allows users to identify a robust set of interactions that maintain or improve model performance in populations with heterogeneous risk, while identifying clinically important effect modification. Robustly selecting two-way interactions will allow developers to avoid stratified models on sparse data, improve model discrimination or calibration, and reduce bias in comparing risk-standardised outcomes across facilities. Based on our results shown here, AHRQ is considering using HGLR to eliminate stratified models in the next iteration of their RA model development.

## Data Availability

The data that support the findings of this study are available from the Agency for Healthcare Research and Quality (AHRQ) through the Healthcare Cost and Utilization Project (HCUP) Central Distributor (https://hcup-us.ahrq.gov/tech_assist/centdist.jsp, email: hcup@ahrq.gov), the California Department of Health Care Access and Information (https://hcai.ca.gov/data-and-reports/research-data-request-information/patient-discharge-data-pdd, email: dataandreports@hcai.ca.gov), and the New York State Department of Health (https://www.health.ny.gov/statistics/sparcs/access, email: sparcs.requests@health.ny.gov). Restrictions apply to the availability of these data, which were used under data use agreements for the current study, and so are not publicly available. Data are however available from the corresponding author (email: mray@ucdavis.edu) upon reasonable request and with permission of the agencies specified above.
